# 161 Be Active Be Well: A community based physical activity behaviour change programme supporting inactive adults to lead more active lives

**DOI:** 10.1093/eurpub/ckae114.007

**Published:** 2024-09-26

**Authors:** Anne Culloty, Clare Deasy

**Affiliations:** Health Service Executive, Ireland

## Abstract

**Purpose:**

Be Active Be Well is a physical activity behaviour change programme for adults who are inactive or not meeting the recommended 150 minutes of moderate intensity physical activity a week. Only 46% of the Irish adult population currently meet this recommendation yet 64% of those inactive want to become more active. Changing and maintaining a health related behaviour is a difficult and complex process. The Be Active Be Well programme is designed to be both supportive and motivational.

The programme embeds evidence based behaviour change theory, strategies and techniques to support participants to explore and understand their physical activity and sedentary behaviours. A key aspect of the programme also includes appropriate signposting to suitable community based physical activity opportunities. The programme also consolidates the impact of Making Every Contact Count (MECC) and chronic disease prevention interventions by enabling participants to understand and lead their physical activity change journey.

**Project description:**

Be Active Be Well was developed and is facilitated by Health Promotion Officers for Physical Activity in Cork Kerry Community Healthcare.

There are 5 sessions in total; each session is 2 hours long. The first four sessions take place once a week over 4 weeks and then 8 weeks later is the 5th and final session. The sessions consist of input from the programme facilitators, group participation, individual reflection and also include practical exercises e.g. stretching, walking, strength and balance.

Evaluation of the initial pilot programme was conducted at the end of each session and at the end of the programme, subsequent programmes are evaluated at the end.

Training for Trainers is planned in order to scale up the programme and expand to all 14 network areas of Cork Kerry Community Healthcare.

**Conclusions:**

Through a multifaceted approach the programme seeks to enable inclusion of those hardest to reach/those who struggle to participate in current community based physical opportunities. Creating links with Social Prescribers, Health Professionals, Family Resource Centres and community groups supports this and ensures recruitment of suitable participants. Providing a supportive environment helps participants to make positive physical activity changes and ultimately improve their overall health and wellbeing.

